# COVID-19 infection and severity among childhood cancer survivors in Denmark and Sweden: a register-based cohort study with matched population and sibling comparisons

**DOI:** 10.1016/j.lanepe.2025.101363

**Published:** 2025-07-04

**Authors:** Javier Louro, Christina-Evmorfia Kampitsi, Hanna Mogensen, Friederike Erdmann, Karin Modig, Anna Nilsson, Mats Heyman, Henrik Hasle, Anja Krøyer, Line Kenborg, Henrik Hjalgrim, Maria Feychting, Giorgio Tettamanti

**Affiliations:** a Unit of Epidemiology, Institute of Environmental Medicine, Karolinska Institutet, Stockholm, Sweden; b Department of Immunology, Genetics and Pathology, Cancer Precision Medicine, Uppsala University, Sweden; c Research group Aetiology and Inequalities in Childhood Cancer, Division of Childhood Cancer Epidemiology, Institute of Medical Biostatistics, Epidemiology and Informatics (IMBEI), University Medical Center of the Johannes Gutenberg University Mainz, Germany; d Department of Prevention and Evaluation, Leibniz Institute for Prevention Research and Epidemiology – BIPS, Bremen, Germany; e Department of Women's and Children's Health, Karolinska Institute, Stockholm, Sweden; f Department of Paediatrics and Adolescent Medicine, Aarhus University Hospital, Aarhus, Denmark; g Childhood Cancer Research Group, Danish Cancer Institute, Danish Cancer Society, Copenhagen, Denmark; h Haematology, Danish Cancer Institute, Danish Cancer Society, Copenhagen, Denmark; i Department of Molecular Medicine and Surgery, Karolinska Institutet, Stockholm, Sweden

**Keywords:** Cancer survival, COVID-19, Neoplasms, Child, Registries

## Abstract

**Background:**

During the COVID-19 pandemic, vulnerable groups faced a higher risk of severe COVID-19 outcomes. The effect of the pandemic on adult childhood cancer survivors is a significant public health concern but not sufficiently understood. We aimed to assess whether adult childhood cancer survivors had a higher risk of severe COVID-19 and registered COVID-19 infections compared to the general population.

**Methods:**

This Nordic register-based cohort study included 5-year childhood cancer survivors diagnosed before age 20 years in Denmark and Sweden, two countries with very different pandemic responses. As comparisons, we randomly selected individuals from the general population, matched on year of birth, sex, and country, and identified all siblings of the survivors. All individuals at least 20 years old and alive on January 1, 2020, were followed until December 31, 2022. We plotted the cumulative hazard rates of severe COVID-19 and registered COVID-19 infection and fitted Cox proportional hazards models estimating adjusted hazard ratios (aHR) with 95% confidence intervals (95% CI).

**Findings:**

The cohort included 13,659 cancer survivors, 58,803 matched comparisons, and 17,531 siblings. Childhood cancer survivors had a lower risk of registered COVID-19 infection (aHR = 0·91; 95% CI = 0·89–0·94) compared to their comparisons but a higher risk of severe COVID-19 (aHR = 1·58; 95% CI = 1·25–1·98). The latter was particularly evident during periods of widespread viral transmission, as reflected in differences between Denmark and Sweden.

**Interpretation:**

These findings underscore the heightened vulnerability of childhood cancer survivors, even many years after their cancer diagnosis, and emphasize the need for closer monitoring and tailored interventions to safeguard this population during future health crises.

**Funding:**

Danish and Swedish Childhood Cancer Foundation, Danish National Research Centre for Childhood Cancer, 10.13039/501100004359Swedish Research Council, and NordForsk.

**Research in context**

**Evidence before this study**

We conducted a systematic literature search in PubMed to identify studies on the risk and prognosis of COVID-19 infection in childhood cancer survivors. Using the query *(COVID-19 [MeSH Terms]) AND (Neoplasms [MeSH Terms]) AND (Child [MeSH Terms]) AND (Survivors [MeSH Terms]) AND (“2020/01/01”[Date–Publication]: “2025/01/01”[Date–Publication])*, we identified a significant gap in the literature. The search yielded 25 studies, however, nearly all focused on psychosocial or lifestyle outcomes. Only one study specifically addressed the risk and prognosis of COVID-19 in childhood cancer survivors. This Canadian study, while relevant, was limited to the province of Ontario, covered only the first 18 months of the pandemic, and reported that just 3% of survivors had tested positive for COVID-19. The authors concluded that there was no evidence of increased risk or worse prognosis in this group but emphasized the need for further research to assess potential risks in older survivors, specific subgroups within the survivor population, and in the context of emerging SARS-CoV-2 variants.

**Added value of this study**

Based on high-quality registry data from Denmark and Sweden, this study is the first European and nation-wide population-based analysis to date on the risk and prognosis of COVID-19 infection in adult childhood cancer survivors. Our findings reveal that, despite a lower risk of COVID-19 infection and higher vaccination rates, childhood cancer survivors faced a higher risk of severe COVID-19 than their matched comparisons. This elevated risk was more pronounced during periods of widespread transmission, such as the outbreak of the pandemic and the emergence of new variants like Alpha at the end of 2020 or Omicron from late 2021. Furthermore, by utilizing data from two Nordic countries with similar healthcare systems but differing pandemic strategies, our study offers a unique opportunity to compare the impact of the pandemic across different settings.

**Implications of all the available evidence**

The population of childhood cancer survivors is steadily growing, highlighting their considerable societal and public health relevance. These survivors remain a vulnerable group even for many years post-cancer diagnosis and would benefit from tailored risk-group interventions during health crises such as the COVID-19 pandemic. The prevalence of severe COVID-19 outcomes was more pronounced among survivors than among comparison groups during periods of high viral spread, confirming a vulnerability to infectious diseases and emphasizing the potential applicability of these findings to more common events like flu season. Additionally, our results indicate that pandemic containment measures in each country may have influenced the extent to which childhood cancer survivors were affected by severe COVID-19.

## Introduction

Globally, over 400,000 children aged 0–19 years are diagnosed with cancer annually.^1^ Improved diagnostics and significant therapeutic progress in recent decades have resulted in notable improvements in survival rates, particularly in high-income countries, where 5-year survival now exceeds 85%.^2^ As a result, the population of childhood cancer survivors continues to grow steadily. However, these survivors represent a vulnerable group, with a markedly higher risk of chronic health conditions and premature mortality compared to the general population.^3^, ^4^, ^5^ Previous studies have reported an increased susceptibility to infectious diseases among childhood cancer survivors, such as influenza and sinopulmonary infections, including pneumonia.^5^^,^^6^ During the COVID-19 pandemic, certain vulnerable groups were identified as being at particularly high risk for severe COVID-19 progression and reduced survival rates including individuals with a history of cancer.^7^ The effect of the COVID-19 pandemic on childhood cancer survivors has, however, not been comprehensively addressed.

Previous research suggested that childhood cancer survivors might be more vulnerable to COVID-19,^8^ but the evidence is inconclusive, and further research is necessary. Gupta et al.^9^ found no increased risk of registered COVID-19 infection or COVID-19-related death among childhood cancer survivors. However, this study was constrained by the short follow-up time, covering only the first half of 2021, with just 3% of the survivors having tested positive for COVID-19. The study called for additional research to understand the pandemic's effects on older childhood cancer survivors and to explore the influence of emerging COVID-19 variants.

The impact of the pandemic on populations varied considerably between countries, as each adopted unique strategies to manage the spread of the SARS-CoV-2 virus. Denmark, for instance, implemented stringent restrictions common across most of Europe, including nationwide lockdown, school closures and mask mandates.^10^ Conversely, Sweden followed a more permissive approach, opting for recommendations rather than mandates and limiting school closures to upper secondary schools, colleges, and universities.^11^ It is unknown if these divergent strategies were particularly relevant in the effect of the pandemic on vulnerable groups such as childhood cancer survivors.

Using data from a long-standing register-based childhood cancer survivor cohort in Denmark and Sweden,^12^ we aimed to assess whether adult childhood cancer survivors had an increased risk of severe COVID-19 and registered COVID-19 infections compared to a matched comparison group from the general population and to their siblings. Moreover, we sought to explore country-specific differences in COVID-19 risk, considering the divergent COVID-19 strategies implemented in Denmark and Sweden.

## Methods

### Study population

We performed a register-based cohort study nested within the Socioeconomic Consequences in Adult Life after Childhood Cancer research program (SALiCCS). All citizens of Denmark and Sweden are assigned a unique personal identification number that enables linkage of individual-level information across various nationwide registries.^13^ Data linkage among those registries was the basis of the SALiCCS research program. Detailed information about the cohort is available elsewhere.^12^

All individuals with a cancer diagnosis (including also non-malignant CNS tumors) at age 19 years or younger were identified using the National Cancer Registers in Denmark and Sweden.^14^ In Sweden, diagnoses were retrieved from 1958 to 2014, and in Denmark, from 1943 to 2008.

A population-based comparison group was established by randomly selecting individuals from the general population, individually matched to survivors by birth year, sex, and country (in Sweden also by municipality) in a 1:5 ratio using incidence density sampling (denoted “comparisons”). The index date for comparisons was defined as the time of cancer diagnosis of their matched case. As an additional comparison group, all siblings of childhood cancer survivors within 10 years of age were identified from the population registers to account for confounding family-shared factors. The index date for siblings was defined as the time when they reached the same age their matched case had at cancer diagnosis. Matched comparisons and siblings diagnosed with cancer before age 20 were excluded from the comparison groups.

Individuals included in the present study had to be at least 20 years old and alive on January 1, 2020 (start of follow-up). They were followed until end of study (December 31, 2022), emigration, or death, whichever came first. Individuals diagnosed with Down syndrome, neurofibromatosis, or tuberous sclerosis complex were excluded to avoid potential confounding. Demographic and vital status information for the childhood cancer survivors, matched comparisons, and siblings was obtained from the Danish Civil Registration System in Denmark and the Total Population Register in Sweden.^13^ Supplementary Figure S1 shows a flowchart of the study population.

### COVID-19 outcomes

Information about COVID-19 diagnoses was collected from the national Danish and Swedish patient registers and causes of death registers.^13^ The patient registers include information on all hospitalizations and specialized outpatient contacts in the two countries. We also obtained information from the Swedish Public Health Agency on all PCR tests for a positive COVID-19 infection (SmiNet) in Sweden^15^ and for all PCR tests (both positive and negative) from Danish Microbiology Database (MiBa) in Denmark.^16^ While a COVID-19 diagnosis from any source (registered COVID-19 infection) depends not only on whether an individual has the disease, but also on the availability of testing and the individuals' propensity to test themselves, hospitalization for or death from COVID-19 likely estimates severe COVID-19 accurately.

### Definition of COVID-19 outcomes

Severe COVID-19 was defined as any overnight hospitalization, admission to intensive care, or death where COVID-19 was the primary diagnosis (Codes DB972A or DB342A in Denmark and ICD = U07·1 or U07·2 in Sweden), or where a respiratory disease (ICD code starting with “J”) was the main diagnosis and COVID-19 was listed as a secondary diagnosis.

Registered COVID-19 infection was defined as a positive PCR test result or an outpatient contact, overnight hospitalization, admission to intensive care, or death in cases where COVID-19 (Codes DB972A or DB342A in Denmark and ICD = U07·1 or U07·2 in Sweden) was listed, in any position, as either a primary or secondary diagnosis.

### Childhood cancer classification

All cancer diagnoses were analyzed both in aggregate and stratified by type, according to the International Classification of Childhood Cancer, Third Edition (ICCC-3). Due to sample size limitations, the main diagnostic groups were categorized as hematological malignancies (including all leukemia subtypes [ICCC-3 Group I] and lymphomas [ICCC-3 Group II]) and solid tumors (including central nervous system [CNS] tumors [ICCC-3 Group III] and non-CNS solid tumors [ICCC-3 Groups IV–XII]).

### Confounders and mediators

We collected information on adjusting covariates of interest, both potential confounders and mediators, from several national registers. A figure illustrating the potential confounders and mediators considered is shown in Supplementary Figure S2.

COVID-19 vaccination was defined as receiving at least one dose of a COVID-19 vaccine during the study period and was categorized as binary (yes/no) irrespective of vaccine type. COVID-19 vaccination data were retrieved from the Danish Vaccination Registry^17^ and the National Vaccination Register in Sweden.^18^

Using the Danish and Swedish patient registers, we obtained information on comorbidities identified in prior literature as risk factors for severe COVID-19,^19^, ^20^, ^21^ including chronic obstructive pulmonary disease (COPD), chronic kidney disease (CKD), diabetes, cardiovascular disease (CVD), and obesity. An individual was considered to have COPD, CKD, or diabetes if they had any inpatient or outpatient care contact related to these conditions between January 1, 2001 and December 31, 2019. For non-chronic comorbidities (obesity and CVD), only health care contacts occurring in the five years preceding the COVID-19 pandemic were considered. Individuals were classified based on the number of comorbidities (0, 1, 2, or 3+). Malignancies diagnosed after age 20 but before January 1, 2020, were also included as an adjustment variable. Information on cancer diagnoses was obtained from the national cancer registers and diagnoses of cancer in situ were excluded.

Highest educational attainment was defined as the highest level of education achieved by the end of 2019 and classified according to the International Standard Classification of Education (ISCED), categorized into three groups: low (primary or lower secondary), medium (upper secondary), and high (postsecondary education). Educational information was obtained from the Longitudinal Integrated Database for Health Insurance and Labor Market Studies (LISA),^22^ and from The Student Registry in Denmark.^23^

### Statistical analysis

The study population were followed from January 1, 2020, until December 31, 2022, or until the earliest occurrence of emigration, death, or the first occurrence of each of the outcomes of interest for each analysis—either a severe COVID-19 diagnosis or a registered COVID-19 infection. We estimated the cumulative hazard rate function using the Nelson–Aalen estimator^24^ and plotted it over the follow-up time. We employed Cox proportional hazards regression models with age as the underlying timescale to estimate adjusted hazard ratios (aHR) with 95% confidence intervals (95% CI) for the first occurrence of each COVID-19 outcome. All models were adjusted for sex, country, highest educational attainment, number of comorbidities, malignancy diagnosed after age 20, and COVID-19 vaccination status. Highest educational attainment, number of comorbidities, malignancy diagnosed after age 20, and COVID-19 vaccination status were considered as potential mediators (Supplementary Figure S2). All models were conducted using complete case analysis. Proportional hazard assumption was assessed testing the independence between the Schoenfeld residuals and time. Covariates that did not meet the proportional hazards assumption were managed using stratified Cox models.^25^ To ensure parsimony, vaccination status was treated as a time-varying covariate, and we assume adherence to the vaccination program and therefore all vaccinated individuals were regarded as fully covered from 14 days after the first vaccination until the end of follow-up.

The main analysis compared childhood cancer survivors to matched comparisons. To assess robustness to shared familial confounding, we replicated all models using siblings as the comparison group, accounting for the correlation between siblings using a robust sandwich estimator. We excluded childhood cancer survivors without siblings from these analyses.

In addition to the overall analysis, we conducted stratified analyses based on the following characteristics: age in 2020 (<50 and ≥50 years old), age at childhood cancer diagnosis or index date (<15 and ≥15 years old), sex (male and female), country (Denmark and Sweden), and time period (January 2020–June 2021 and July 2021–December 2022). Adjustment was applied as for the main analysis, except for the specific stratification variable.

All analyses were performed in R version 4·3·1.^26^

### Ethics approval

Ethical approval was granted from the Stockholm Regional Ethics Committee and the Swedish Ethical Review Authority (2016/25-31/5, 2022/01281-02) and the study is registered in the Danish Cancer Institute's internal project database (2018-DCRC-0044) in agreement with the General Data Protection Regulation. The study protocol for this study has been published on clinicaltrials.gov (NCT06482281).

### Role of the funding source

The funding source had no role in study design; in the collection, analysis, and interpretation of data; in the writing of the report; or in the decision to submit the paper for publication.

## Results

### Characteristics of the study population

The analytical sample included 13,659 childhood cancer survivors, of whom 10,906 had siblings, alongside 58,803 matched comparisons and 17,531 sibling comparisons (Table 1 and Supplementary Figure S1). The mean age at baseline (January 1, 2020) was 40·8 years for the childhood cancer survivors, 40·0 for the matched comparisons, and 40·8 for the siblings. Approximately 70% of participants were from Sweden. Comorbidities were more common among survivors than comparisons and siblings, and a higher proportion of childhood cancer survivors had taken at least one COVID-19 vaccination (90·3% vs 88·8%, Table 1). The proportion of individuals with severe COVID-19 was 0·8% among survivors (n = 110), 0·4% among matched comparisons (n = 240), and 0·4% among siblings (n = 77), and did not differ between countries except for siblings (0·3% of siblings in Denmark and 0·5% in Sweden, Supplementary Table S1). A registered COVID-19 infection was more frequent among comparisons and siblings than among childhood cancer survivors (Table 1) and was higher in Denmark than in Sweden among both survivors, comparisons, and siblings (Supplementary Table S1). A timeline showing the chronology of major COVID-19-related events in both Denmark and Sweden is provided in Supplementary Figure S3.

**Table 1:** Characteristics of childhood cancer survivors, matched comparisons, and siblings.

	Survivors	Matched comparisons	Siblings
	N = 13,659	N = 58,803	N = 17,531
Age at 2020, mean (sd)	40·8 (14·2)	40·0 (13·8)	40·8 (13·4)
Time since diagnosis at 2020, mean (sd)	29·7 (14·1)	–	–
Country, n (%)			
Denmark	4425 (32·4)	17,640 (30·0)	4923 (28·1)
Sweden	9234 (67·6)	41,163 (70·0)	12,608 (71·9)
Sex, n (%)			
Male	7226 (52·9)	31,127 (52·9)	8965 (51·1)
Female	6433 (47·1)	27,676 (47·1)	8566 (48·9)
Age at index date, n (%)			
0–14 years	8645 (63·3)	37,301 (63·4)	11,196 (63·9)
15–19 years	5014 (36·7)	21,502 (36·6)	6335 (36·1)
Childhood cancer classification, n (%)			
Hematological malignancies	4542 (33·3)	–	–
Solid tumors	9117 (66·5)	–	–
Highest educational attainment, n (%)			
Low	1958 (14·3)	6966 (11·8)	2148 (12·3)
Medium	5991 (43·9)	27,109 (46·1)	8175 (46·6)
High	5547 (40.6)	24,306 (41·3)	7107 (40·5)
Missing	163 (1·2)	422 (0·7)	101 (0·6)
Number of comorbidities, n (%)			
0	10,957 (80·2)	52,024 (88·5)	15,409 (87·9)
1	2153 (15·8)	5692 (9·7)	1756 (10·0)
2	454 (3·3)	896 (1·5)	293 (1·7)
≥3	95 (0·7)	191 (0·3)	73 (0·4)
Malignancy diagnosed after age 20, n (%)			
No	12,521 (91·7)	56,908 (96·8)	16,890 (96·3)
Yes	1138 (8·3)	1895 (3·2)	641 (3·7)
At least one vaccination, n (%)			
No	1321 (9·7)	6585 (11·2)	1943 (11·1)
Yes	12,338 (90·3)	52,218 (88·8)	15,588 (88·9)
Severe COVID-19 infection, n (%)			
No	13,549 (99·2)	58,563 (99·6)	17,454 (99·6)
Yes	110 (0·8)	240 (0·4)	77 (0·4)
Registered COVID-19 infection, n (%)			
No	8628 (63·2)	35,854 (61·0)	10,722 (61·2)
Yes	5031 (36·8)	22,949 (39·0)	6809 (38·8)

### Cumulative hazard rate of severe COVID-19 and registered COVID-19 infection

**Fig. 1:**
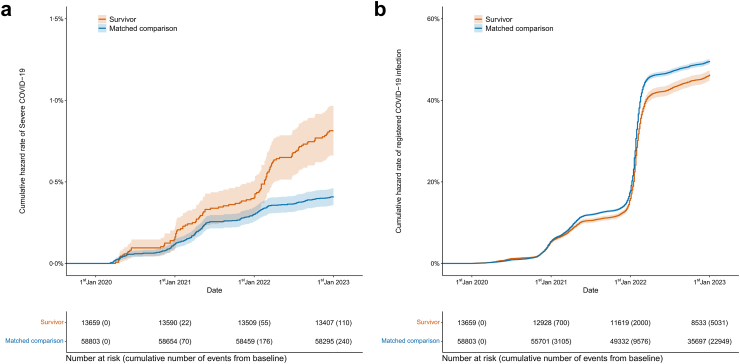
Cumulative hazard rate COVID-19 though the follow-up. **(a)** Cumulative hazard rate of severe COVID-19. **(b)** Cumulative hazard rate of registered COVID-19 infection.

In the first months of the pandemic, the cumulative hazard rate of severe COVID-19 was similar for childhood cancer survivors and matched comparisons, although survivors had a slightly higher cumulative hazard rate during late spring 2020 (Fig. 1a). At the start of 2021, as COVID-19 infections surged with the dominance of the Alpha variant, the cumulative hazard rate of severe COVID-19 increased more among survivors than among comparisons. This divergence became even more pronounced from early 2022 with the widespread transmission of the Omicron variant. In contrast, among the comparisons, the increase in the cumulative hazard rate of severe COVID-19 during this period was much less pronounced, leading to a significant divergence between the cumulative hazard rate curves of survivors and comparisons (Fig. 1a).

On the other hand, the cumulative hazard rate of registered COVID-19 infection was higher in matched comparisons than in childhood cancer survivors (Fig. 1b), with the initial divergence of cumulative hazard rate curves around mid-2021. With the spread of the Omicron variant, there was a steep increase in the cumulative hazard rate of registered COVID-19 infection in both groups in early 2022, but the increase was more pronounced among the comparisons. Exact numbers regarding severe COVID-19 and registered COVID-19 infection can be found in Supplementary Table S2.

**Fig. 2:**
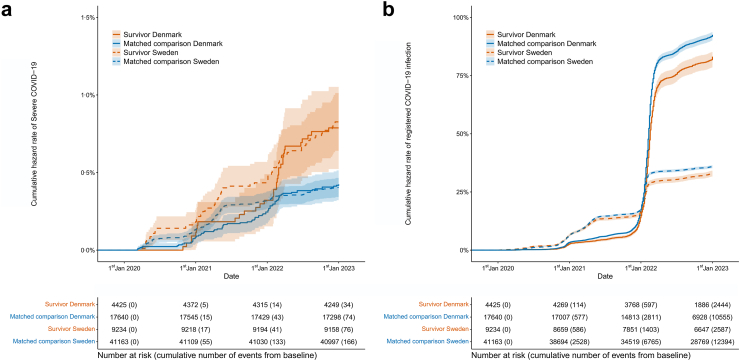
Cumulative hazard rate COVID-19 though the follow-up stratified by country. **(a)** Cumulative hazard rate of severe COVID-19. **(b)** Cumulative hazard rate of registered COVID-19 infection.

In country-specific analyses, the cumulative hazard rate of severe COVID-19 in Denmark was similar among childhood cancer survivors and their matched comparisons until the beginning of 2022 (Fig. 2a) and remained consistently lower than in Sweden. In Sweden, the hazard rate was already higher among survivors than comparisons during the initial spread of COVID-19 in 2020. This difference further widened with the emergence of the Alpha variant at the beginning of 2021. From early 2022, a clear divergence between childhood cancer survivors and comparisons emerged in both countries, with survivors experiencing a steeper increase of severe COVID-19 than comparisons. Notably, the cumulative hazard rates of severe COVID-19 among Danish survivors and comparisons rose considerably at this time, reaching the Swedish levels in early 2022 and maintaining both countries at the same level until the end of the follow-up. At the end of the study period, i.e., 31 December 2022, 0·79% of survivors in Denmark and 0·83% in Sweden had been diagnosed with severe COVID-19. Corresponding proportions for the comparisons were 0·42% and 0·40% in Denmark and Sweden, respectively. Exact numbers are given in Supplementary Table S3.

The cumulative hazard rate of registered COVID-19 infection remained relatively similar between survivors and their matched comparisons within each country until 2022 (Fig. 2b). From 2022 onward, a noticeable divergence emerged, with higher cumulative rates among comparisons than survivors in both countries. The estimated cumulative hazard rate of registered COVID-19 infection is not directly comparable between countries, as COVID-19 testing was substantially more accessible in Denmark than in Sweden throughout the pandemic.

Despite the smaller sample size, the cumulative hazard rate results were similar for both severe COVID-19 and registered COVID-19 infection when comparing childhood cancer survivors with siblings instead of matched comparisons. Survivors had a higher cumulative hazard rate of severe COVID-19 and lower of registered COVID-19 infection than siblings (Supplementary Figure S4).

### Risk of severe COVID-19

**Fig. 3:**
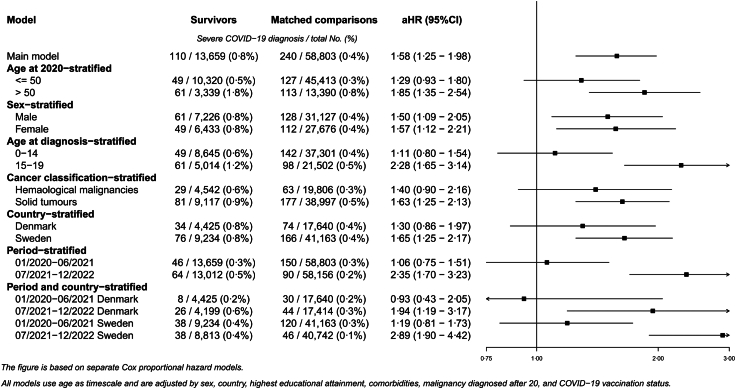
Adjusted hazard ratios (aHR) of severe COVID-19 comparing childhood cancer survivors with population comparisons.

The higher risk of severe COVID-19 among childhood cancer survivors compared to their matched comparisons is also evident from the adjusted Cox proportional hazards regression models (aHR = 1·58; 95% CI = 1·25–1·98) (Fig. 3). Stratified analyses indicated a more pronounced risk increase in survivors 50 years or older at baseline (aHR = 1·85; 95% CI = 1·35–2·54); in Sweden (aHR = 1·65; 95% CI = 1·25–2·17); in survivors from solid tumors (aHR = 1·63; 95% CI = 1·25–2·13); and among survivors diagnosed with cancer at age 15 years or older (aHR = 2·28; 95% CI = 1·65–3·14). During the first period of the pandemic, January 2020–June 2021 the hazard ratio was close to unity, whereas in the second period (July 2021–December 2022), the risk of severe COVID-19 was more than two times higher in survivors compared to their matched comparisons (aHR = 2·35; 95% CI = 1·70–3·23). This period-specific increased risk was more pronounced in Sweden (aHR = 2·89; 95% CI = 1·90–4·42) than in Denmark (aHR = 1·94; 95% CI = 1·19–3·17). Results did not differ notably between men and women.

### Risk of registered COVID-19 infection

**Fig. 4:**
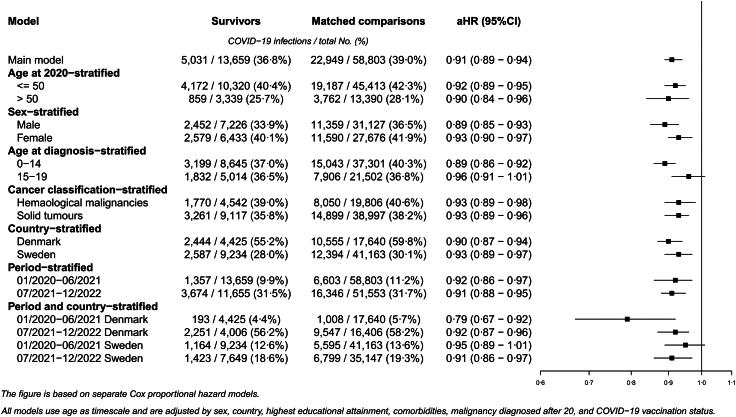
Adjusted hazard ratios (aHR) of registered COVID-19 infection comparing childhood cancer survivors with population comparisons.

Childhood cancer survivors had a lower risk of registered COVID-19 infection compared to their matched comparisons (aHR = 0·91; 95% CI = 0·89–0·94) (Fig. 4). This finding was consistent in all stratified analyses, except during the first period, in which the reduced risk was more pronounced in Denmark (aHR = 0·79; 95% CI = 0·67–0·92) than in Sweden (aHR = 0·95; 95% CI = 0·89–1·01), with no difference between countries during the second period.

Although the statistical power was lower, similar results for both severe COVID-19 and registered COVID-19 infection risk were observed when comparing childhood cancer survivors to their siblings (Supplementary Figures S5 and S6). Crude hazard ratios for both risk of severe COVID-19 and registered COVID-19 infection are given in Supplementary Table S4.

## Discussion

This study used unique and high-quality data from administrative and health registers for 13,659 childhood cancer survivors, 58,803 matched comparisons, and 17,531 sibling comparisons from Denmark and Sweden. The findings reveal that although childhood cancer survivors had a lower risk of attaining COVID-19, and a higher vaccination rate, they had a 1·6-fold higher risk of developing severe COVID-19 compared to their matched comparisons and to their siblings. The increased risk was particularly pronounced among survivors diagnosed with childhood cancer at age 15 years or older and survivors aged 50 and older at the start of the pandemic. The higher risk was especially evident during periods of widespread viral transmission, such as the initial outbreak of COVID-19, the emergence of new strains like Alpha, or when Omicron became the dominant variant.

Our findings regarding severe COVID-19 align with previous research documenting increased susceptibility to infections^5^^,^^6^ among childhood cancer survivors. Our results are also consistent with Magone et al. who described how vulnerable populations, including survivors of adult cancer, experienced worse COVID-19 outcomes.^7^ However, our finding of a consistently lower risk of registered COVID-19 infection among childhood cancer survivors compared to their matched comparisons contrasts with findings from Gupta et al.,^9^ who reported no such difference in infection risk during the pandemic's initial 18 months. This discrepancy may be attributed to differences in the study periods, as the lower risk of registered COVID-19 among survivors was particularly pronounced with the Omicron wave, which was not included in Gupta et al.'s analysis, as well as differences in the setting, given that their study was conducted solely in the region of Ontario, Canada. This finding may also reflect more precautions taken by survivors to avoid infection.

Although the absolute risk remained low for both childhood cancer survivors and the general population, the risk of severe COVID-19 among survivors diverged from that of the general population during key periods of the pandemic: the initial outbreak, early 2021, and early 2022. These periods coincide with the highest transmission rates and the emergence of major variants—Alpha and Omicron.^27^^,^^28^ In contrast, the cumulative hazard rate difference between childhood cancer survivors and the general population remained more stable during periods when the incidence of COVID-19 was better controlled, such as the latter half of 2020 and 2021, and in Denmark until the end of 2021. These findings highlight the increased vulnerability of this group during high-transmission periods, suggesting they could have benefited from being classified as high-risk and having tailored interventions, like being prioritized when COVID-19 vaccines became available. We highlight that older childhood cancer survivors (aged 50 years old or older), survivors diagnosed with cancer at older ages, and survivors diagnosed with solid tumors, may represent especially vulnerable populations, as we observed a greater risk of severe COVID-19 in these groups.

Hazard ratios were consistently higher in Sweden than in Denmark, even though by the end of the study period the proportion of childhood cancer survivors with a diagnosis of severe COVID-19 was similar in both countries, and strongest associations were found during widespread viral transmission. Pandemic management strategies differed considerably between the countries; Denmark followed the more widely adopted European model, implementing stringent public health measures early on during the pandemic, including lockdowns, school closures, mask mandates and other social restrictions.^10^ Meanwhile, Sweden adopted a more lenient, advisory approach.^11^ From February 1st, 2022, Denmark lifted all restrictions. COVID-19 vaccines became available in both countries at the end of 2020, providing protection especially against severe disease. Vaccines have been shown to be less effective in immunocompromised individuals,^29^ which may also have influenced the higher risk of severe COVID-19 among survivors.

It is important to interpret the differences between the countries cautiously, as estimates of registered COVID-19 may differ as Denmark conducted more tests than Sweden.^30^ Nevertheless, within-country comparisons clearly demonstrate a higher risk of severe COVID-19 among survivors at times of widespread viral transmission. Importantly, analyses were adjusted for risk factors known to be associated with severe COVID-19, which should therefore not have affected the comparison between childhood cancer survivors and population comparisons.

The generalizability of these findings to other high-income countries depends on several factors, including healthcare system similarities, vaccination rates, and pandemic management strategies. Given that childhood cancer survivors in Denmark and Sweden had high vaccination rates, their relatively low risk of severe COVID-19 may not be as evident in countries with lower vaccination coverage. In regions where vaccine uptake is lower—both among the general population and vulnerable groups like childhood cancer survivors—, the increased relative risk of severe COVID-19 in childhood cancer survivors compared with the general population could be even more pronounced. Differences in healthcare access and public health responses may further influence outcomes, suggesting that caution is needed when extrapolating these results to other countries.

The present study utilized data from two Nordic countries with similar healthcare systems but different pandemic strategies and a different pattern of COVID-19 transmission in the population, providing a unique opportunity to assess the impact of the pandemic. All our data was sourced from population-based registers, which offer nearly complete information with virtually no loss to follow-up. As a result, our study was not affected by the information bias often associated with self-reported data, nor by selection bias due to non-participation of specific survivor subgroups. In addition, having information from siblings allowed us to perform a sensitivity analysis whose consistent results add robustness to our findings.

However, some limitations warrant consideration. Control sampling design on the cohort differs between Denmark and Sweden, as was performed without replacement in Denmark and with replacement in Sweden. However, we believe that the differences in controls sampling in the two countries did not have any impact on the results. We did not have information on lifestyle factors, such as smoking, physical activity, and BMI that could affect the risk of severe COVID-19. Nevertheless, we expect these characteristics to act as mediators rather than confounders, and thus, we do not anticipate that our findings were significantly altered. Another limitation concerns the lack of statistical power, which prevented stratification by specific diagnostic groups, an approach that would have been valuable due to the considerable differences in cancer therapy and the associated risk of somatic late effects. However, we were able to stratify cancers as hematological malignancies versus solid tumors, and this analysis showed no substantial differences, lending robustness to our findings. Similarly, sensitivity analyses using siblings often lacked statistical significance, despite maintaining the direction of the results, due to the lower number of siblings compared to the number of matched comparisons. Furthermore, the limited statistical power required grouping the comorbidities identified as potential mediators into a single variable, potentially limiting the ability to fully adjust for them in the analysis. Additionally, information on comorbidities was based on hospitalizations and contacts with specialized outpatient care; therefore, primary care contacts were not captured which may lead to misclassification of some individuals, possibly in a differential manner between cancer survivors and comparisons. Finally, we considered individuals vaccinated if they had one dose of vaccine. If childhood cancer survivors had a different pattern of repeated doses and therefore more protected, our observed association would be somewhat underestimated. However, the high vaccination adherence observed in both countries likely mitigates this concern.

This study provides valuable insights into the COVID-19 pandemic that help shape strategies in the event of future pandemics or other health emergencies. Despite having a lower registered COVID-19 infection rate, adult childhood cancer survivors had a 58% higher risk of severe COVID-19 compared to the general population, highlighting their vulnerability even many years after surviving their childhood cancer. The difference between survivors and comparisons became more pronounced during periods of widespread viral transmission, information that may be crucial for establishing protocols to protect vulnerable populations in future pandemics or even during periods of high incidence of infectious diseases such as the flu season.

### Conclusion

Although childhood cancer survivors had a lower risk of registered COVID-19 infection and a higher vaccination rate compared to their matched comparisons, they faced a 58% increased risk of severe COVID-19. The increased risk was especially pronounced during periods of widespread viral transmission in the general population. These findings highlight the heightened vulnerability of childhood cancer survivors and emphasize the need to consider this group as at higher risk, proposing tailored interventions to safeguard this population during future health crises.

## Contributors

JL, FE, MF, and GT were responsible for the conceptualization and methodology of the study. JL, CEK, HM, MF, and GT contributed to the visualization of the study. JL, AK, and GT performed the data curation. MF and GT contributed with the supervision of the article and the funding acquisition. FE, HM, LK, AK, MF, and GT were involved in the data collection. HM, LK, MF, and GT were responsible for the project administration. JL performed the formal analysis and wrote the original draft, and JL, CEK, HM, FE, KM, AN, MH, HHa, AK, LK, HHj, MF, and GT critically reviewed, edited the manuscript, and approved the final manuscript before submission.

## Data sharing statement

The data analyzed in this study are remotely stored in a secure platform at Statistics Denmark. Pseudonymized personal data were acquired from national registry authorities following ethical approval (where applicable) and secrecy assessment. Danish and Swedish laws and regulations do not allow sharing of personal sensitive data, which can only be made available for researchers who fulfill the legal requirements for access to such data. Please contact Line Kenborg (kenborg@cancer.dk) with inquiries regarding data access.

## Declaration of interests

The authors declare no conflict of interest.
