# Supplementary material for: COVID-19 infection and severity among childhood cancer survivors in Denmark and Sweden: a register-based cohort study with matched population and sibling comparisons
Source: Lancet Reg Health Eur. 2025 Jul 4;55:101363. doi: 10.1016/j.lanepe.2025.101363 (PMC12426850; doi:10.1016/j.lanepe.2025.101363)
Supplement: Supplementary Figures and Tables [file mmc1.pdf]

**Supplementary figure 1. Flowchart of the study population.**

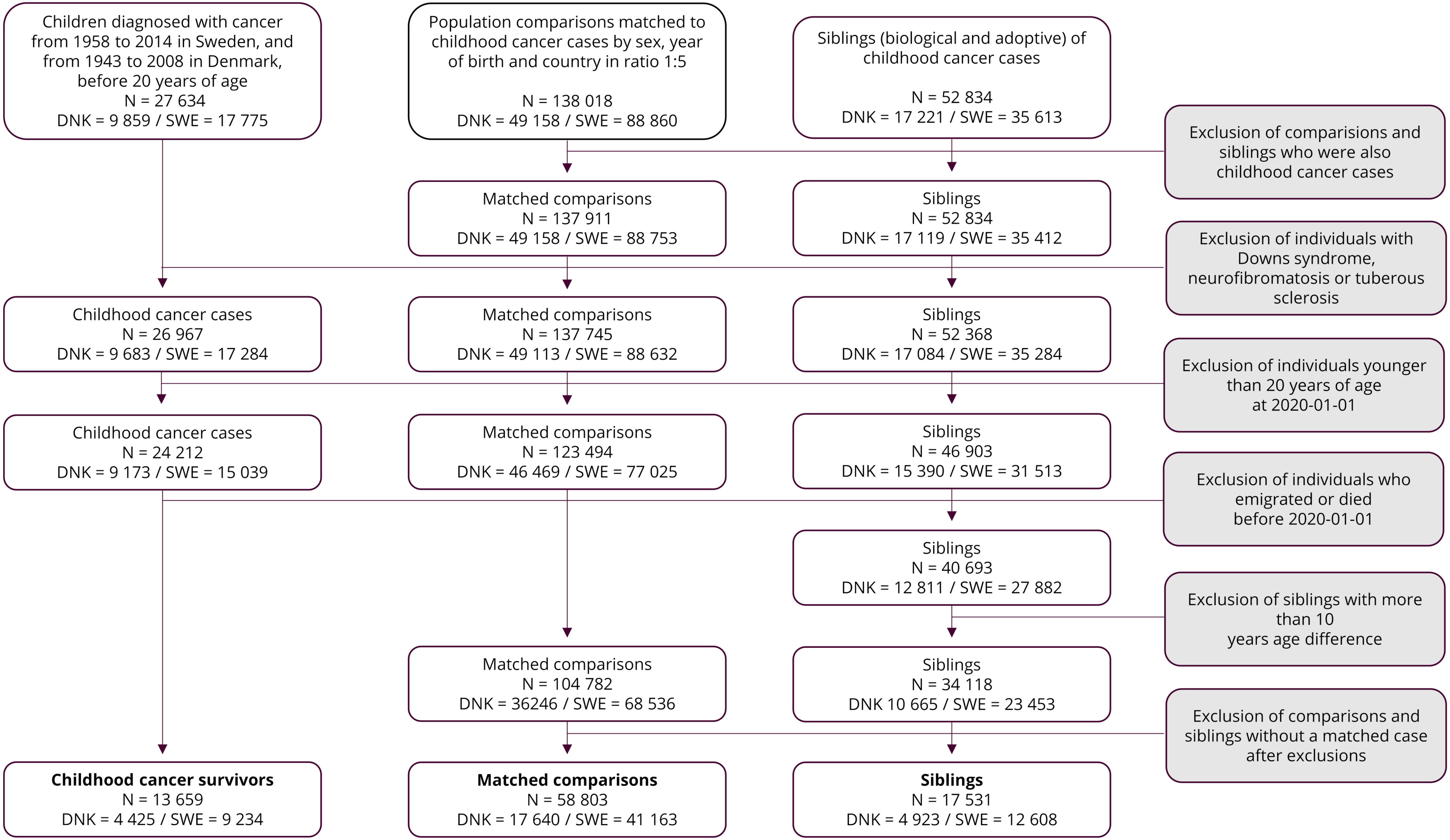

**Supplementary figure 2. Figure illustrating the potential confounders and mediators between "Being a childhood cancer survivor in 2020" (E) and "Registered COVID-19 infection and Severe COVID-19 diagnosis" (D).**

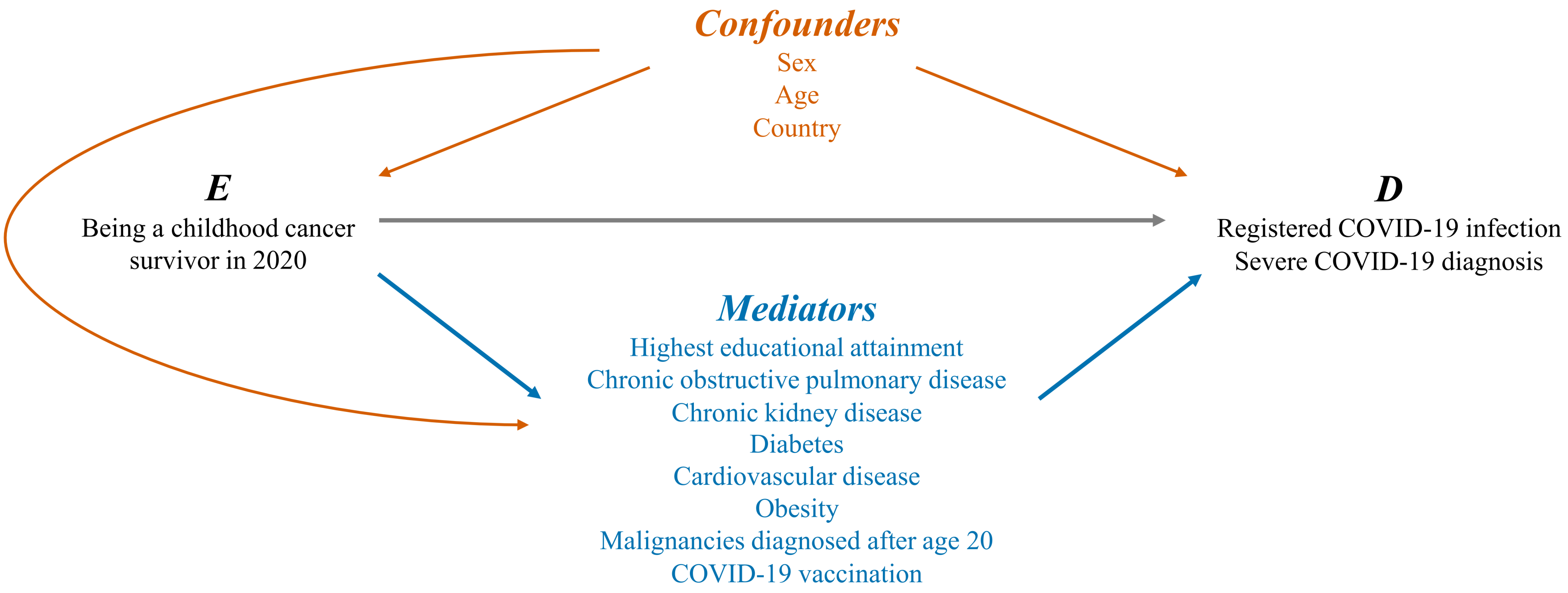

**Supplementary figure 3. Chronology of major COVID-19 related events in Denmark and Sweden between January 2020 and January 2022.**

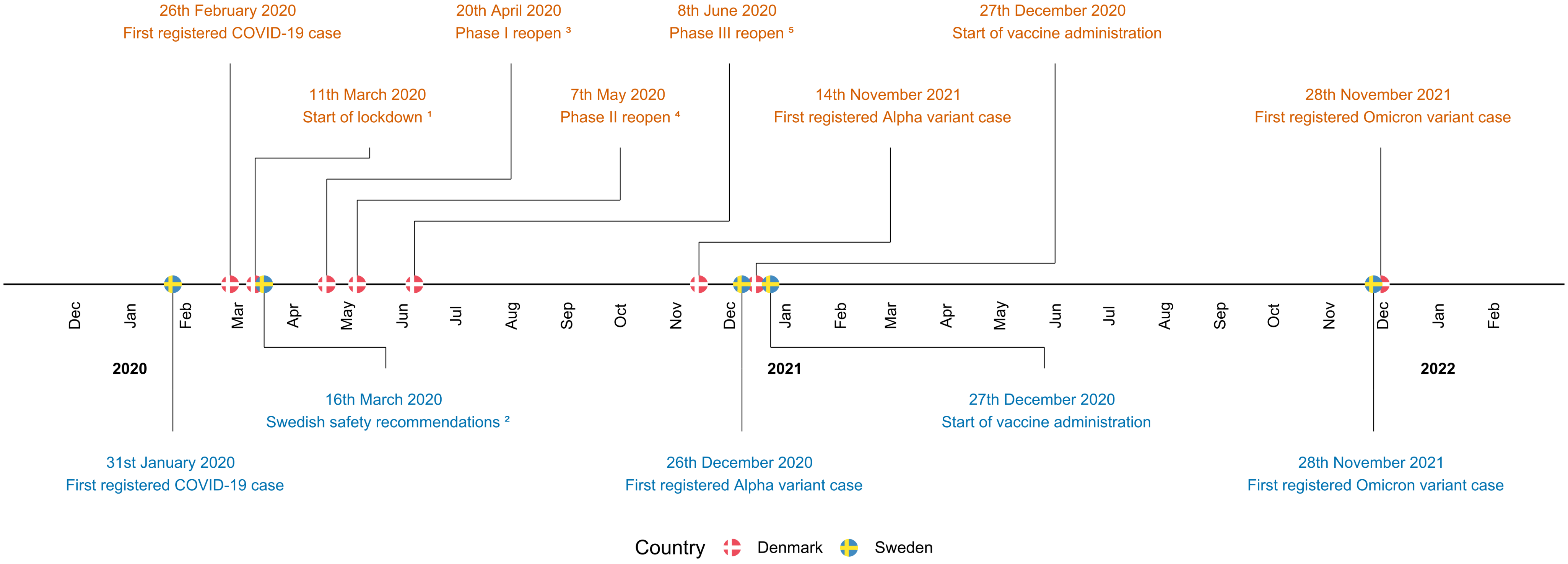


[1] 11th of March. The prime minister of Denmark announces in a press conference that Denmark is temporarily shut down. Schools, institutions and day care facilities are closed. Ban on gatherings of more than 100 people. Public servants who do not have a critical function are sent home. One week later the prohibition against gatherings will be reduced to 10 people and, in addition, hairdressers, restaurants, and shopping centers will close.

[2] 16th of March. The Public Health Agency of Sweden recommends that education in upper secondary schools, municipal adult education, vocational higher education and university education should be provided via remote or distance teaching, a recommendation that was also followed by high schools.

[3] 20th of April. Driving schools, hairdressers, research laboratories and certain other liberal professions as well as the youngest grade levels and outdoor sports without physical contact reopen in Denmark. One week later the courts will reopen.

[4] 7th of May. All outdoor sports and association life, professional sports without spectators and zoos where guests transport themselves around by car reopen in Denmark. Shopping centres, arcades, department stores and the like will be allowed to reopen 4 days later. Churches, religious communities, restaurants, cafes, libraries, museums, cinemas, zoos and theaters will reopen the following days.

[5] 8th of June. Fitness centers, swimming pools and amusement parks reopen.The assembly ban is lifted from 10 to 50 people, and will be lifted lifted from 50 to 100 people one month later.

**Supplementary figure 4. Cumulative hazard rate of severe COVID-19 and registered COVID-19 infection using siblings as comparisons.**

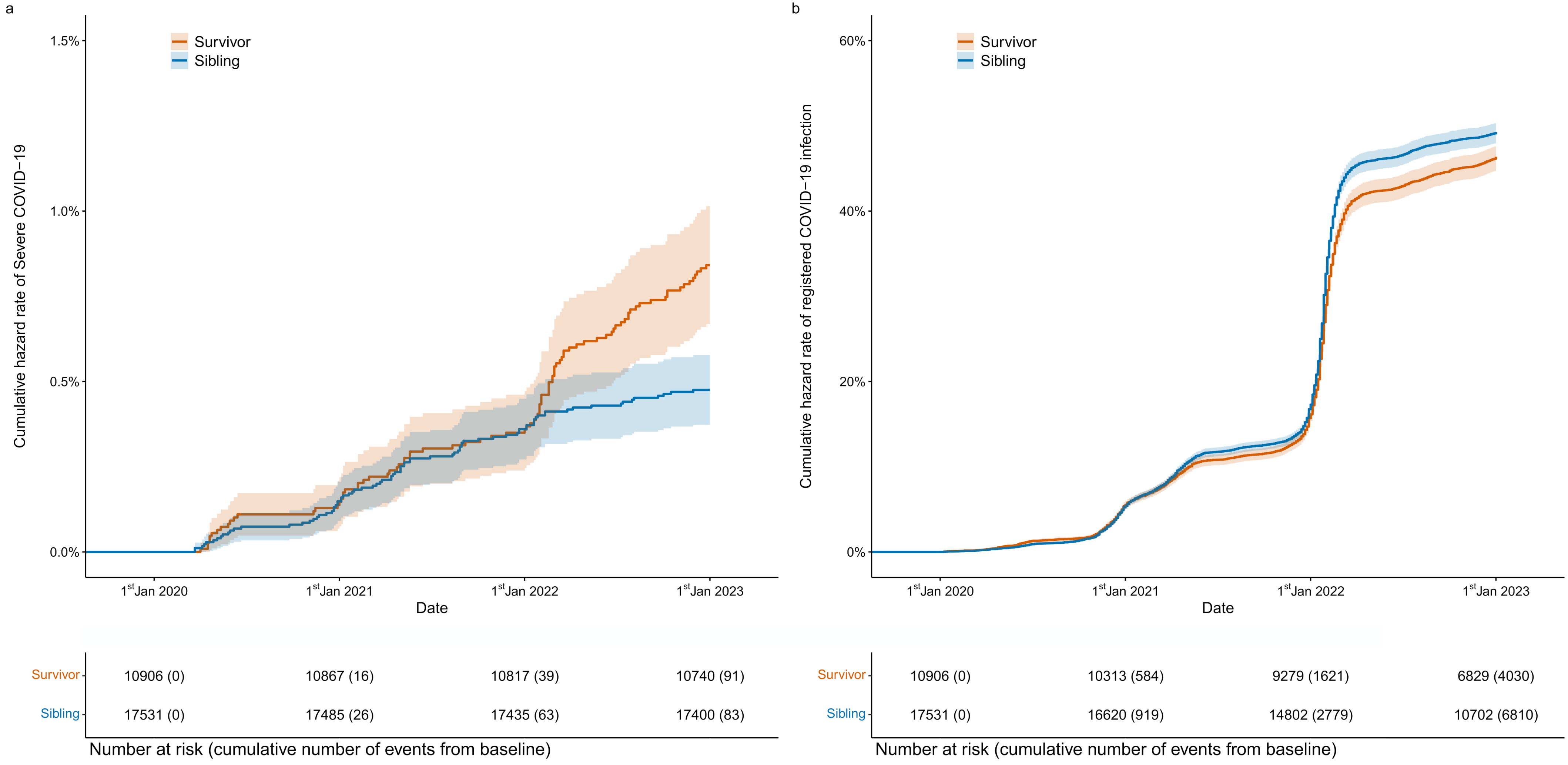

**Supplementary figure 5. Adjusted hazard ratios of severe COVID-19 using siblings as comparisons.**

| Model | Survivors | Siblings | aHR (95%CI) |
|---|---|---|---|
| | *Severe COVID−19 diagnosis / total No. (%)* | | |
| Main model | 81 / 10,906 (0·7%) | 77 / 17,531 (0·4%) | 1·60 (1·16 − 2·21) |
| **Age at 2020−stratified** | | | |
| <= 50 | 41 / 8,490 (0·5%) | 36 / 13,054 (0·3%) | 1·33 (0·86 − 2·07) |
| > 50 | 40 / 2,416 (1·7%) | 41 / 4,477 (0·9%) | 1·83 (1·18 − 2·85) |
| **Sex−stratified** | | | |
| Male | 47 / 5,759 (0·8%) | 41 / 8,965 (0·5%) | 1·68 (1·11 − 2·54) |
| Female | 34 / 5,147 (0·7%) | 36 / 8,566 (0·4%) | 1·46 (0·89 − 2·38) |
| **Age at diagnosis−stratified** | | | |
| 0−14 | 40 / 6,961 (0·6%) | 44 / 11,196 (0·4%) | 1·28 (0·83 − 1·98) |
| 15−19 | 41 / 3,945 (1·0%) | 33 / 6,335 (0·5%) | 1·97 (1·25 − 3·09) |
| **Cancer classification−stratified** | | | |
| Haematological malignancies | 25 / 3,688 (0·7%) | 22 / 5,874 (0·4%) | 1·58 (0·92 − 2·71) |
| Solid tumours | 56 / 7,218 (0·8%) | 55 / 11,657 (0·5%) | 1·64 (1·12 − 2·41) |
| **Country−stratified** | | | |
| Denmark | 17 / 3,256 (0·5%) | 14 / 4,923 (0·3%) | 1·64 (0·78 − 3·43) |
| Sweden | 64 / 7,650 (0·8%) | 63 / 12,608 (0·5%) | 1·62 (1·13 − 2·32) |
| **Period−stratified** | | | |
| 01/2020−06/2021 | 33 / 10,906 (0·3%) | 49 / 17,531 (0·3%) | 1·23 (0·77 − 1·95) |
| 07/2021−12/2022 | 48 / 10,259 (0·5%) | 28 / 16,884 (0·2%) | 2·71 (1·65 − 4·45) |
| **Period and country−stratified** | | | |
| 01/2020−06/2021 Denmark | <5 / 3,256 (<0·2%) | 6 / 4,923 (0·1%) | 0·49 (0·09 − 2·58) |
| 07/2021−12/2022 Denmark | 12−17 / 3,030 (~0·8%) | 8 / 4,697 (0·2%) | 2·56 (0·99 − 6·60) |
| 01/2020−06/2021 Sweden | 28−33 / 7,650 (~0·4%) | 43 / 12,608 (0·3%) | 1·24 (0·78 − 1·97) |
| 07/2021−12/2022 Sweden | 31−36 / 7,229 (~0·5%) | 20 / 12,187 (0·2%) | 2·39 (1·34 − 4·25) |

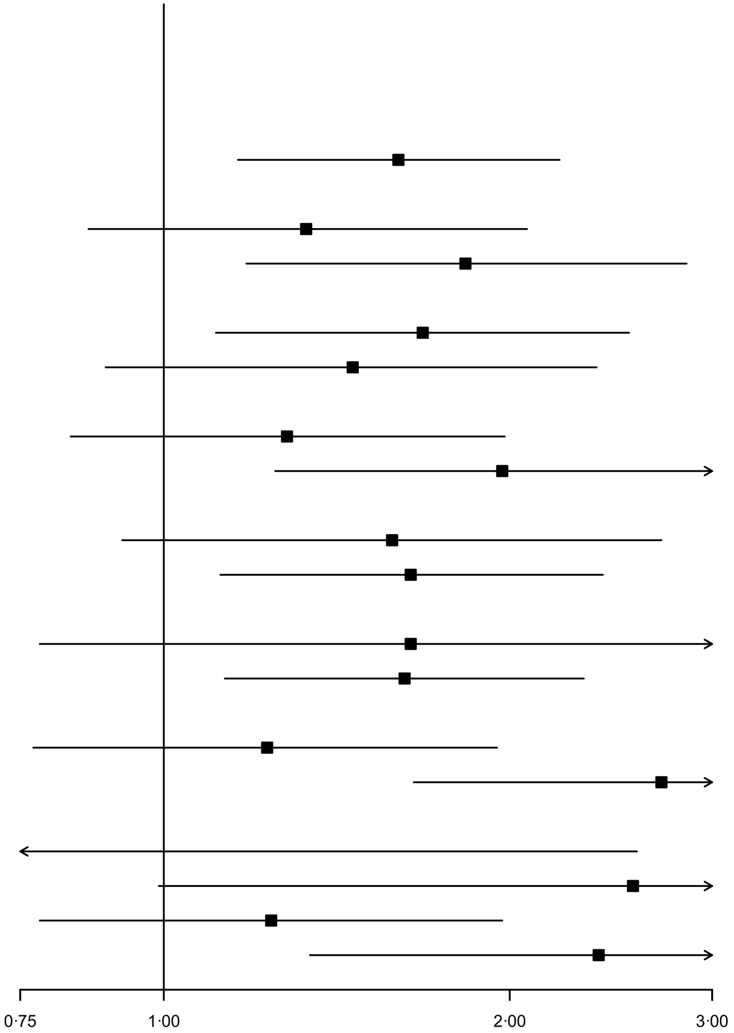


*The figure is based on separate Cox proportional hazard models.*

*All models use age as timescale and are adjusted by sex, country, highest educational attainment, comorbidities, malignancy diagnosed after 20, and COVID−19 vaccination status.*

**Supplementary figure 6. Adjusted hazard ratios of registered COVID-19 infection using siblings as comparisons.**

| Model | Survivors | Matched comparisons | aHR (95%CI) |
|---|---|---|---|
| | *COVID−19 infections / total No. (%)* | | |
| Main model | 4,030 / 10,906 (37·0%) | 6,809 / 17,531 (38·8%) | 0·91 (0·88 − 0·95) |
| **Age at 2020−stratified** | | | |
| <= 50 | 3,409 / 8,490 (40·2%) | 5,601 / 13,054 (42·9%) | 0·91 (0·87 − 0·94) |
| > 50 | 621 / 2,416 (25·7%) | 1,208 / 4,477 (27·0%) | 0·94 (0·86 − 1·03) |
| **Sex−stratified** | | | |
| Male | 1,953 / 5,759 (33·9%) | 3,102 / 8,965 (34·6%) | 0·91 (0·86 − 0·96) |
| Female | 2,077 / 5,147 (40·4%) | 3,707 / 8,566 (43·3%) | 0·92 (0·87 − 0·96) |
| **Age at diagnosis−stratified** | | | |
| 0−14 | 2,580 / 6,961 (37·1%) | 4,447 / 11,196 (39·7%) | 0·89 (0·85 − 0·93) |
| 15−19 | 1,450 / 3,945 (36·8%) | 2,362 / 6,335 (37·3%) | 0·93 (0·87 − 0·99) |
| **Cancer classification−stratified** | | | |
| Haematological malignancies | 1,446 / 3,688 (39·2%) | 2,379 / 5,874 (40·5%) | 0·95 (0·89 − 1·01) |
| Solid tumours | 2,584 / 7,218 (35·8%) | 4,430 / 11,657 (38·0%) | 0·90 (0·86 − 0·95) |
| **Country−stratified** | | | |
| Denmark | 1,858 / 3,256 (57·1%) | 3,024 / 4,923 (61·4%) | 0·91 (0·86 − 0·95) |
| Sweden | 2,172 / 7,650 (28·4%) | 3,785 / 12,608 (30·0%) | 0·92 (0·87 − 0·97) |
| **Period−stratified** | | | |
| 01/2020−06/2021 | 1,115 / 10,906 (10·2%) | 1,940 / 17,531 (11·1%) | 0·93 (0·87 − 1·00) |
| 07/2021−12/2022 | 2,915 / 9,144 (31·9%) | 4,869 / 14,944 (32·6%) | 0·91 (0·87 − 0·95) |
| **Period and country−stratified** | | | |
| 01/2020−06/2021 Denmark | 134 / 3,256 (4·1%) | 274 / 4,923 (5·6%) | 0·76 (0·62 − 0·93) |
| 07/2021−12/2022 Denmark | 1,724 / 2,896 (59·5%) | 2,750 / 4,423 (62·2%) | 0·91 (0·86 − 0·97) |
| 01/2020−06/2021 Sweden | 981 / 7,650 (12·8%) | 1,666 / 12,608 (13·2%) | 0·96 (0·89 − 1·03) |
| 07/2021−12/2022 Sweden | 1,191 / 6,248 (19·1%) | 2,119 / 10,521 (20·1%) | 0·89 (0·82 − 0·95) |

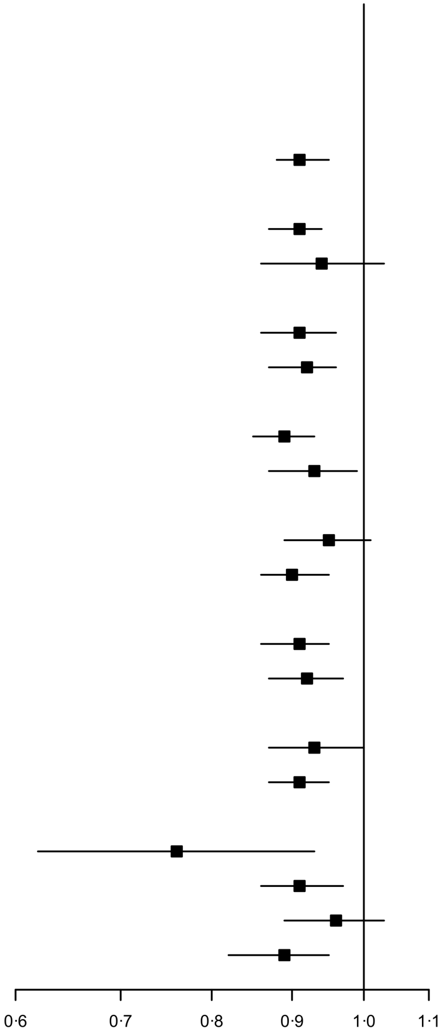


*The figure is based on separate Cox proportional hazard models.*

*All models use age as timescale and are adjusted by sex, country, highest educational attainment, comorbidities, malignancy diagnosed after 20, and COVID−19 vaccination status.*

**Supplementary table 1. Comparison of the basic characteristics of the overall study populations in Denmark and Sweden.**

| | Denmark | | | Sweden | | |
|---|---|---|---|---|---|---|
| | **Survivors** | **Matched comparisons** | **Siblings** | **Survivors** | **Matched comparisons** | **Siblings** |
| | **N = 4,425** | **N = 17,640** | **N = 4,923** | **N = 9,234** | **N = 41,163** | **N = 12,608** |
| Age at 2020, mean (sd) | 41·7 (14·7) | 40·5 (14·0) | 39·7 (11·9) | 40·3 (14·0) | 39·8 (13·7) | 41·3 (14·0) |
| Time since diagnosis at 2020, mean (sd) | 31·1 (14·5) | 29·9 (13·8) | 29·2 (12·1) | 29·0 (13·9) | 28·4 (13·8) | 30·0 (14·1) |
| Sex (%) | | | | | | |
| Male | 2,455 (55·5) | 9,782 (55·5) | 2,538 (51·6) | 4,771 (51·7) | 21,345 (51·9) | 6,427 (51·0) |
| Female | 1,970 (44·5) | 7,858 (44·5) | 2,385 (48·4) | 4,463 (48·3) | 19,818 (48·1) | 6,181 (49·0) |
| Age at index date, n (%) | | | | | | |
| 0-14 | 2,953 (66·7) | 11,956 (67·8) | 3,315 (67·3) | 5,692 (61·6) | 25,345 (61·6) | 7,881 (62·5) |
| 15-19 | 1,472 (33·3) | 5,684 (32·2) | 1,608 (32·7) | 3,542 (38·4) | 15,818 (38·4) | 4,727 (37·5) |
| Cancer classification, n (%) | | | | | | |
| Haematological malignancies | 1,477 (33·4) | 6,054 (34·3) | 1,698 (34·5) | 3,065 (33·2) | 13,752 (33·4) | 4,176 (33·1) |
| Solid tumours | 2,948 (66·6) | 11,586 (65·7) | 3,225 (65·5) | 6,169 (66·8) | 27,411 (66·6) | 8,432 (66·9) |
| Highest educational attainment, n (%) | | | | | | |
| Low | 1,011 (22·9) | 3,093 (17·7) | 871 (17·8) | 947 (10·4) | 3873 (9·5) | 1,277 (10·2) |
| Medium | 1,707 (38·7) | 7,348 (42·1) | 2,071 (42·2) | 4,284 (47·1) | 19,761 (48·3) | 6,104 (48·7) |
| High | 1,688 (38·3) | 7,010 (40·2) | 1,965 (40·0) | 3,859 (42·5) | 17,296 (42·3) | 5,142 (41·1) |
| Missing | 19 (0·4) | 189 (1·1) | 16 (0·3) | 144 (1·6) | 233 (0·6) | 85 (0·7) |
| Number of comorbidities, n (%) | | | | | | |
| 0 | 3,507 (79·3) | 15,211 (86·2) | 4,318 (87·7) | 7,450 (80·7) | 36,813 (89·4) | 11,091 (88·0) |
| 1 | 743 (16·8) | 2,045 (11·6) | 519 (10·5) | 1,410 (15·3) | 3,647 (8·9) | 1,237 (9·8) |
| 2 | 146 (3·3) | 312 (1·8) | 67 (1·4) | 308 (3·3) | 584 (1·4) | 226 (1·8) |
| >= 3 | 29 (0·7) | 72 (0·4) | 19 (0·4) | 66 (0·7) | 119 (0·3) | 54 (0·4) |
| Malignancy diagnosed after age 20, n (%) | | | | | | |
| No | 3,943 (89·1) | 16,872 (95·6) | 4,731 (96·1) | 8,578 (92·9) | 40,036 (97·3) | 12,159 (96·4) |
| Yes | 482 (10·9) | 768 (4·4) | 192 (3·9) | 656 (7·1) | 1127 (2·7) | 449 (3·6) |
| At least one vaccination, n (%) | | | | | | |
| No | 386 (8·7) | 1,961 (11·1) | 468 (9·5) | 935 (10·1) | 4,624 (11·2) | 1,475 (11·7) |
| Yes | 4,039 (91·3) | 15,679 (88·9) | 4,455 (90·5) | 8,299 (89·9) | 36,539 (88·8) | 11,133 (88·3) |
| Severe COVID-19 infection, n (%) | | | | | | |
| No | 4,391 (99·2) | 17,566 (99·6) | 4,909 (99·7) | 9,158 (99·2) | 40,997 (99·6) | 12,545 (99·5) |
| Yes | 34 (0·8) | 74 (0·4) | 14 (0·3) | 76 (0·8) | 166 (0·4) | 63 (0·5) |
| Registered COVID-19 infection, n (%) | | | | | | |
| No | 1,981 (44·8) | 7,085 (40·2) | 1,899 (38·6) | 6,647 (72·0) | 28,769 (69·9) | 8,823 (70·0) |
| Yes | 2,444 (55·2) | 10,555 (59·8) | 3,024 (61·4) | 2,587 (28·0) | 12,394 (30·1) | 3,785 (30·0) |

**Supplementary table 2.Cumulative hazard rate of severe COVID-19 and registered COVID-19 infection using matched comparisons.**

| | Severe COVID-19 | | Registered COVID-19 infection | |
|---|---|---|---|---|
| | **Survivors** | **Matched comparision** | **Survivors** | **Matched comparision** |
| 1st July 2020 | 0·10 (0·04-0·15) | 0·06 (0·04-0·08) | 1·15 (0·97-1·33) | 0·85 (0·77-0·92) |
| 1st January 2021 | 0·15 (0·08-0·21) | 0·12 (0·09-0·14) | 5·15 (4·77-5·54) | 5·30 (5·11-5·49) |
| 1st July 2021 | 0·34 (0·24-0·44) | 0·26 (0·21-0·30) | 10·50 (9·94-11·06) | 11·93 (11·64-12·22) |
| 1st January 2022 | 0·40 (0·29-0·50) | 0·30 (0·26-0·34) | 15·46 (14·78-16·15) | 17·30 (16·95-17·65) |
| 1st July 2022 | 0·68 (0·54-0·82) | 0·37 (0·32-0·41) | 42·66 (41·43-43·89) | 46·86 (46·24-47·49) |
| 1st January 2023 | 0·81 (0·66-0·97) | 0·41 (0·36-0·46) | 46·03 (44·75-47·32) | 49·50 (48·85-50·15) |

Each cell in the table shows the cumulative hazard rate per 100 people and the 95% confidence interval.

**Supplementary table 3. Cumulative hazard rate of severe COVID-19 and registered COVID-19 infection using matched comparisons and stratified by country.**

| | Severe COVID-19 | | | |
|---|---|---|---|---|
| | **Survivors Denmark** | **Matched comparision Denmark** | **Survivors Sweden** | **Matched comparision Sweden** |
| 1st July 2020 | 0·00 (0·00-0·00) | 0·02 (0·00-0·04) | 0·14 (0·06-0·22) | 0·08 (0·05-0·10) |
| 1st January 2021 | 0·09 (0·00-0·18) | 0·08 (0·04-0·12) | 0·17 (0·09-0·26) | 0·13 (0·10-0·17) |
| 1st July 2021 | 0·18 (0·06-0·31) | 0·17 (0·11-0·23) | 0·41 (0·28-0·54) | 0·29 (0·24-0·34) |
| 1st January 2022 | 0·32 (0·15-0·49) | 0·25 (0·17-0·32) | 0·43 (0·30-0·57) | 0·32 (0·27-0·38) |
| 1st July 2022 | 0·72 (0·46-0·97) | 0·38 (0·29-0·48) | 0·66 (0·50-0·83) | 0·36 (0·30-0·42) |
| 1st January 2023 | 0·79 (0·52-1·05) | 0·42 (0·32-0·51) | 0·83 (0·64-1·01) | 0·40 (0·34-0·47) |

Each cell in the table shows the cumulative hazard rate per 100 people and the 95% confidence interval.

| | Registered COVID-19 infection | | | |
|---|---|---|---|---|
| | **Survivors Denmark** | **Matched comparision Denmark** | **Survivors Sweden** | **Matched comparision Sweden** |
| 1st July 2020 | 0·25 (0·10-0·40) | 0·20 (0·14-0·27) | 1·58 (1·33-1·84) | 1·13 (1·02-1·23) |
| 1st January 2021 | 2·52 (2·04-2·99) | 3·25 (2·98-3·52) | 6·43 (5·90-6·95) | 6·19 (5·94-6·43) |
| 1st July 2021 | 4·51 (3·87-5·15) | 5·91 (5·55-6·28) | 13·47 (12·70-14·25) | 14·61 (14·23-14·99) |
| 1st January 2022 | 13·92 (12·77-15·06) | 16·62 (15·99-17·25) | 16·23 (15·37-17·08) | 17·60 (17·18-18·03) |
| 1st July 2022 | 75·50 (72·34-78·67) | 85·19 (83·47-86·91) | 30·36 (29·15-31·58) | 34·19 (33·57-34·81) |
| 1st January 2023 | 81·84 (78·49-85·20) | 91·88 (90·06-93·70) | 32·87 (31·60-34·15) | 35·82 (35·19-36·46) |

Each cell in the table shows the cumulative hazard rate per 100 people and the 95% confidence interval.

**Supplementary table 4. Unadjusted hazard ratios for severe COVID-19 and registered COVID-19 infection.**

| | **Severe COVID-19** | | **Registered COVID-19 infection** | |
|---|---|---|---|---|
| **Model** | **Survivors vs matched comparisons** | **Survivors vs siblings** | **Survivors vs matched comparisons** | **Survivors vs siblings** |
| **Main model** | 1·88 (1·50-2·36) | 1·81 (1·32-2·47) | 0·93 (0·90-0·96) | 0·92 (0·88-0·95) |
| **Age at 2020-stratified** | | | | |
| <=50 | 1·67 (1·20-2·32) | 1·72 (1·10-2·69) | 0·93 (0·90-0·96) | 0·91 (0·87-0·94) |
| >50 | 2·11 (1·55-2·88) | 1·90 (1·22-2·96) | 0·91 (0·85-0·98) | 0·94 (0·86-1·03) |
| **Sex-stratified** | | | | |
| Male | 1·92 (1·42-2·61) | 1·98 (1·30-3·00) | 0·91 (0·87-0·95) | 0·94 (0·89-0·99) |
| Female | 1·82 (1·30-2·55) | 1·64 (1·02-2·65) | 0·95 (0·91-0·99) | 0·90 (0·86-0·95) |
| **Age at diagnosis-stratified** | | | | |
| 0-14 | 1·44 (1·04-2·00) | 1·55 (1·01-2·38) | 0·90 (0·87-0·93) | 0·89 (0·85-0·93) |
| 15-19 | 2·52 (1·83-3·47) | 2·12 (1·34-3·36) | 0·99 (0·94-1·04) | 0·95 (0·90-1·01) |
| **Cancer classification-stratified** | | | | |
| Haematological malignancies | 1·93 (1·25-2·99) | 1·97 (1·12-3·46) | 0·94 (0·89-0·98) | 0.94 (0.88-0.99) |
| Solid tumours | 1·86 (1·43-2·42) | 1·76 (1·21-2·57) | 0·92 (0·89-0·96) | 0.90 (0.86-0.95) |
| **Country-stratified** | | | | |
| Denmark | 1·71 (1·14-2·56) | 1·93 (0·94-3·96) | 0·90 (0·87-0·93) | 0·89 (0·85-0·93) |
| Sweden | 1·97 (1·50-2·58) | 1·77 (1·25-2·51) | 0·92 (0·88-0·96) | 0·91 (0·86-0·96) |
| **Period-stratified** | | | | |
| 01/2020-06/2021 | 1·26 (0·90-1·75) | 1·19 (0·77-1·85) | 0·90 (0·85-0·95) | 0·91 (0·85-0·98) |
| 07/2021-12/2022 | 2·96 (2·15-4·08) | 2·93 (1·82-4·72) | 0·95 (0·92-0·98) | 0·92 (0·88-0·96) |
| **Period and country-stratified** | | | | |
| 01/2020-06/2021 Denmark | 0·99 (0·45-2·14) | 0·58 (0·12-2·70) | 0·78 (0·67-0·91) | 0·74 (0·60-0·90) |
| 07/2021-12/2022 Denmark | 2·25 (1·38-3·66) | 2·83 (1·17-6·84) | 0·91 (0·87-0·95) | 0·89 (0·84-0·94) |
| 01/2020-06/2021 Sweden | 1·35 (0·94-1·94) | 1·32 (0·83-2·09) | 0·94 (0·88-1·00) | 0·96 (0·89-1·03) |
| 07/2021-12/2022 Sweden | 3·58 (2·33-5·50) | 2·89 (1·64-5·11) | 0·91 (0·86-0·97) | 0·89 (0·83-0·95) |

Each cell in table shows the unadjusted hazard ratio and the 95% confidence interval.
